# COMMON UNMET NEEDS OF DEMENTIA CAREGIVERS AND HOW THESE DIFFER BY DISEASE STAGE AND RACE

**DOI:** 10.1093/geroni/igz038.2027

**Published:** 2019-11-08

**Authors:** Quincy M Samus, Danetta Sloan, Jeannie-Marie Leoutsakos, Betty Black, Deirdre Johnston

**Affiliations:** The Johns Hopkins University, Baltimore, Maryland, United States

## Abstract

This presentation (co-presentation Samus and Sloan) will use combined cross-sectional, baseline data from two intervention studies (n=-642) conducted in Maryland evaluating the impact of dementia care coordination model (MIND at home) to provide a detailed description of common modifiable unmet care needs of family caregivers of community-living persons with dementia (PWD), explore how care needs may differ by the disease stage of the PWD, and presence of racial disparities in care needs. Unmet caregiver needs were identified based on comprehensive in-home assessments using a standardized tool (JHDCNA 2.0) with 6 care domains and 18 items covering caregiver needs. Family caregivers were 77% women; 63 years old (mean); 60% White; and 52% adult children. The most prevalent needs were for education/resources (98%), legal (74%), mental health (44%), and informal support (43%). Needs varied based on dementia severity. African American caregivers (vs. white) had significantly more unmet caregiver needs at baseline (p<.001).

